# *Plasmodium falciparum* reticulocyte-binding homologues are targets of human inhibitory antibodies and play a role in immune evasion

**DOI:** 10.3389/fimmu.2025.1532451

**Published:** 2025-03-25

**Authors:** Linda Reiling, Kristina E. M. Persson, Fiona J. McCallum, Nimmo Gicheru, Samson M. Kinyanjui, Chetan E. Chitnis, Freya J. I. Fowkes, Kevin Marsh, James G. Beeson

**Affiliations:** ^1^ Department of Life Sciences, Burnet Institute of Medical Research and Public Health, Melbourne, VIC, Australia; ^2^ Department of Medicine, University of Melbourne, VIC, Australia; ^3^ Department of Immunology, Monash University, Melbourne, VIC, Australia; ^4^ Department of Laboratory Medicine, Lund University, Lund, Sweden; ^5^ Clinical Chemistry and Pharmacology, Skåne University Hospital, Lund, Sweden; ^6^ Australian Defence Force Malaria and Infectious Disease Institute, Enoggera, QLD, Australia; ^7^ Centre for Geographic Medicine Research (Coast), Kenya Medical Research Institute - Wellcome Trust Research Programme, Kilifi, Kenya; ^8^ Department of Parasites and Insect Vectors, Pasteur Institute, Paris, France; ^9^ Centre for Epidemiology and Biostatistics, Melbourne School of Population and Global Health, The University of Melbourne, Melbourne, VIC, Australia; ^10^ Department of Epidemiology and Preventive Medicine, Monash University, Melbourne, VIC, Australia; ^11^ Nuffield Department of Medicine, Centre for Clinical Vaccinology and Tropical Medicine, University of Oxford, Oxford, United Kingdom; ^12^ Department of Microbiology, Monash University, Melbourne, VIC, Australia; ^13^ School of Translational Medicine, Monash University, Melbourne, VIC, Australia; ^14^ Department of Infectious Diseases, University of Melbourne, Melbourne, VIC, Australia

**Keywords:** *P. falciparum*, inhibitory antibodies, phenotypic variation, immune evasion, reticulocyte binding homologues, RH proteins

## Abstract

**Introduction:**

Antibodies targeting the blood-stage of *Plasmodium falciparum* play a critical role in naturally acquired immunity to malaria by limiting blood-stage parasitemia. One mode of action of antibodies is the direct inhibition of merozoite invasion of erythrocytes through targeting invasion ligands. However, evasion of inhibitory antibodies may be mediated in *P. falciparum* by switching between various ligand-mediated merozoite invasion pathways. Here, we investigated the potential roles of invasion ligands PfRH1, PfRH2a and PfRH2b in immune evasion through phenotypic variation, and their importance as targets of human invasion-inhibitory antibodies.

**Methods:**

Serum samples from malaria-exposed children and adults in Kenya were examined for their ability to inhibit *P. falciparum* invasion, using parasites with disrupted pfrh1, pfrh2a or pfrh2b genes.

**Results and Discussion:**

The loss of PfRH1 and PfRH2b substantially impacted on susceptibility to inhibitory antibodies, suggesting that variation in the use of these ligands contributes to immune evasion. The effect was less prominent with loss of PfRH2a. Differential inhibition of the knockout and parental lines points to PfRH1 and PfRH2b as targets of acquired growth inhibitory antibodies whereas PfRH2a appeared to be a minor target. There was limited relatedness of the inhibitory responses between different isolates or compared to parasites with deletions of erythrocyte-binding antigens. This further suggests that there is a substantial amount of antigenic diversity in invasion pathways to facilitate immune evasion. These findings provide evidence that PfRH1 and PfRH2b are significant targets of inhibitory antibodies and variation in their expression may facilitate immune evasion. Targeting of multiple invasion ligands in vaccine design is likely to be required to achieve potent inhibitory antibodies and protective efficacy against malaria.

## Introduction

Despite recent advances, malaria is an ongoing major global health problem with approximately 249 million cases in 85 endemic countries and an estimated 608,000 deaths per year in 2022. *Plasmodium falciparum* is responsible for the majority of malaria-attributable morbidity and mortality and has the ability to cause repeated infections through evasion of the host’s immune response (1–4). Naturally acquired immunity against the blood stage of infection occurs over time after repeated exposure, and is thought to prevent clinical symptoms by controlling blood-stage parasitemia (5, 6). Various proteins on the merozoite surface and in the apical organelles (7, 8) are targets of naturally acquired antibodies and are associated with protection from clinical malaria (9–13). Effective immunity may require responses to multiple antigens (7, 14). To date, achieving substantial efficacy with vaccines based on merozoite antigens has been challenging. Recent progress in understanding molecular events involved in host cell infection may provide new approaches for the development of vaccines based on merozoite antigens (15) (4).

*P. falciparum* merozoite invasion of red blood cells (RBCs) involves several steps, including initial attachment of the parasite to the surface of the RBC, reorientation, tight-junction formation, and finally, penetration of the RBC membrane (16). Two protein families and their respective membrane receptors have been demonstrated to play important roles in the invasion of RBCs: the *Plasmodium falciparum* reticulocyte-binding homolog (PfRH) proteins and the erythrocyte binding antigens (EBAs) (17). These interactions occur early in the process of invasion, after initial merozoite attachment and, therefore, are an attractive vaccination target (16, 17).

Clinical immunity is likely to result from multiple effector mechanisms of anti-merozoite antibodies (7, 18–23), including direct anti-invasion ligand antibodies to PfRHs and EBAs (10–12, 24). Antibodies that target invasion ligands to inhibit invasion are thought to be important contributors to immunity, and generating these inhibitory antibodies has been a major focus of blood-stage vaccine development (15). However, our knowledge about specific targets of invasion-inhibitory antibodies remains limited. Furthermore, *P. falciparum* is able to use alternate invasion pathways to evade inhibitory antibodies by expressing and/or using different PfRHs and EBAs, a process known as phenotypic variation (25, 26). This impacts the acquisition of immunity and strategies for vaccine development.

PfRHs are a family of invasion ligands located in the rhoptries, and the roles of different members of this family have been defined to various degrees. All the members of the PfRH family (except PfRH2a) bind directly to the surface of RBCs through specific receptors (27–31), but the functional relevance of these interactions is yet to be fully determined (1). The lesser characterized PfRH1 binds to a neuraminidase-sensitive, unidentified receptor Y (32–34). PfRH2 exists in two forms, PfRH2a and PfRH2b, but only PfRH2b is thought to bind to the neuraminidase-resistant but as yet unidentified receptor Z (1). PfRH4 binds to complement receptor 1 (CR1) on the erythrocyte surface (35). Thus far, PfRH5 [which lacks a transmembrane domain, unlike other PfRH members (27)] is considered the only PfRH protein essential for invasion and not involved in phenotypic variation (36). PfRH5 has progressed to be the most-promising blood-stage vaccine candidate to date and induces inhibitory antibodies in natural infection and human vaccination trials (37–40). However, in a human challenge study, PfRH5 antibodies only resulted in a moderate reduction of the parasite multiplication rate (38), suggesting that PfRH5 may need to be combined with another antigen to achieve more potent immunity.

Monoclonal antibodies against both PfRH1 and PfRH2b suppress intracellular Ca2+ signaling, resulting in a lack of EBA175 surface expression and the inhibition of junction formation required for invasion (34, 41). Affinity-purified human antibodies against PfRH4 are able to limit parasite growth *in vitro* (11). More recent work suggests that PfRH proteins are part of the moving junction as PfRH1 proteolytic fragments have been co-localized in close proximity to AMA1 (42). PfRH2b has also been located in the moving junction during merozoite invasion (41).

In addition to invasion inhibition, members of the PfRH protein family have been identified as targets of functional antibodies that induce other downstream effector functions such as complement fixation and opsonic phagocytosis. Importantly, the presence of these functional antibodies in clinical samples was strongly associated with protection from febrile illness (43, 44). However, our knowledge about the relative contribution and importance of each functional antibody response in acquired immunity is still incomplete. The EBA family of invasion ligands comprises EBA140, EBA175, EBA181, and EBL1, which bind directly to the erythrocyte surface antigens Glycophorin C, Glycophorin A, the erythrocyte protein 4.1, and Glycophorin B, respectively (45–48). Like PfRH antigens, EBAs are targets of inhibitory antibodies, and switching invasion pathway ligands has been linked to immune evasion (26).

In naturally acquired immunity, antibodies against various merozoite surface antigens and invasion ligands are co-acquired, so dissection of the relative functional importance of antibodies in individuals living in malaria endemic areas is challenging. *P. falciparum* strain-specific preferences for different invasion pathways and antigen polymorphisms further impede standard approaches. The use of *P. falciparum* lines with targeted deletion or modification of specific invasion proteins in invasion inhibition assays offers one technical solution to investigate the role of antigen-specific inhibitory antibodies, as has been demonstrated for EBAs (25, 26).

In this study, we hypothesized that PfRH1 and PfRH2 are targets of acquired immunity and that the variation in expression and/or utilization of these invasion proteins facilitates evasion of inhibitory antibodies. To investigate this, we evaluated the functional growth inhibition activity of acquired antibodies from malaria-exposed individuals against *P. falciparum* lines with a single deletion of PfRH1, PfRH2a, or PfRH2b, compared to parental *P. falciparum*. We investigated the relationship and the overlap between inhibitory antibodies to different PfRH and EBA targets and the relationship between inhibitory antibodies and antibodies to recombinant antigens quantified by enzyme-linked immunosorbent assay (ELISA).

## Materials and methods

### Study population

Ethical approval was obtained from the Ethics Committee of the Kenya Medical Research Institute, Nairobi, Kenya, and from the Walter and Eliza Hall Institute Ethics Committee, Melbourne, Australia. All samples were obtained following written informed consent.

Samples were selected from a number of transmission settings over time from Kilifi County as described and used in previous studies (25, 26). The aim was to include samples from subjects with diverse malaria exposure and to enable comparisons between children and adults. Cohort 1 was comprised of sera samples that were randomly selected from a community-based cross-sectional survey of residents (total n=148) in Ngerenya in Kilifi County, Kenya, in 1998, a year that was preceded by a relatively high incidence of malaria in the region (49). Samples from 19 adults [median age (range) of 47 (18–81) years, 22.2% male, 15.8% *P. falciparum* positive] and 52 children [median age (range) of 8 (2–12) years, 60.4% male, 48.1% *P. falciparum* positive] were selected randomly from this survey for use in growth inhibition assays comparing 3D7 wild-type and 3D7ΔRH1 (n=71).

As a comparison, Cohort 2 comprised 31 samples randomly selected from children participating in a cohort study (Ngerenya, Kenya, collected in 2003) during a year that was preceded by less malaria than in 1998. These samples [median age of 5 (0-8) years, 41.9% male, 12.9% *P. falciparum* positive) were selected from a larger cohort based on their EBA inhibitory activity after first screening them for growth inhibition against *P. falciparum* 3D7 wild-type, and, as previously tested, for growth inhibition of *P. falciparum* with deletion of EBA175, EBA140, and EBA181 (25, 26). All 31 samples were tested in growth inhibition assays comparing 3D7 wild-type and 3D7ΔRh1. However, due to limited sample volumes, only 17 of these 31 samples from the Ngerenya 2003 cohort were tested against 3D7ΔRH2a and 3D7ΔRH2b.

Cohort 3 comprised randomly selected sera from 29 anonymous adult blood donors (age, sex, and parasitemic status unknown) collected in May 2004 in Kilifi. These additional samples were selected due to their growth-inhibitory effect on 3D7 that had been described in previous assays (25, 26). They had also been used in growth inhibition assays comparing 3D7 wild-type and 3D7ΔRH1. Due to insufficient sample volume, only 22 of these samples were tested on 3D7ΔRH2a and 3D7ΔRH2b (see **Supplementary Table S1**). A total of n=148 samples from Cohort 1 were used in the ELISA to quantify the presence of PfRH1 and PfRH2 specific antibodies. Of these, a subset (n=71) was used in GIAs; n=31 samples and n=28 samples from Cohorts 2 and 3, respectively, were also used in GIAs.

As previously described, samples from the above cohorts had been tested for growth inhibition of *P. falciparum* with the deletion of EBA175, EBA140, and EBA181 (25, 26), and relevant data are included here for comparison. Sera from malaria-naïve naïve non-exposed adult residents in Australia and the UK were used as negative controls.

### Enzyme-linked immunosorbent assay

ELISAs were performed as described elsewhere (10) with the following recombinantly expressed proteins: recombinant PfRH1 and PfRH2-2030 antigens, which were expressed and purified as described previously (10, 50). Briefly, amino acids 2030-2528, covering a part of the common region of PfRH2a and PfRH2b, were expressed as GST fusion protein PfRH2-2030 in *Escherichia coli* and purified on glutathione agarose (Sigma Alderich) following the manufacturers’ instructions. After elution, the recombinant protein was dialyzed overnight into HTPBS. The erythrocyte-binding region of PfRH1 (amino acids 500-833) was expressed in *E. coli* as a hexa-6-His fusion protein from a codon optimized gene (GeneArt) as described previously (50) and was kindly provided by Chetan E. Chitnis.

For the ELISAs, antigen was coated overnight onto 96-well microtitre flat bottom plates (Maxisorp, Nunc) at 1µg/ml in PBS. Blocking was performed using 10% skim milk in PBS/0.05% Tween20 for 2 hours at 37˚C, and all subsequent washes were performed with the latter solution. Sera were diluted to a final concentration of 1/500 using 5% skim milk in PBS/0.05% Tween20 and incubated for 2 hours at room temperature. Incubation with HRP conjugated goat anti-human IgG antibody (Millipore) followed for 1 hour at room temperature at 1/5,000 in 5% skim milk/0.05% Tween20. ABTS liquid substrate (Sigma) was used to visualize the enzymatic reaction and stopped with 1% SDS. Absorbance was read at 405nm. All samples were performed in duplicate, and results were excluded if the discrepancy was ≥25% between duplicates. Sero-positive samples from exposed individuals and samples from unexposed Melbourne donors were included as positive and negative controls on each plate for validation and standardization purposes.

### *P. falciparum* culture

*P. falciparum*-infected erythrocytes were grown at 2% hematocrit in RPMI-HEPES culture media supplemented with 50µg/ml hypoxanthine, 25mM NaHCO_3_, 20µg/ml Gentamicin, 2µg/ml L-Glutamine, 5% (vol/vol) heat-inactivated pooled human sera from donors resident in Australia, and 0.25% Albumax (Gibco, Invitrogen), and were maintained in an atmosphere of 1% O_2_, 4% CO_2_ and 95% N_2_ at 37˚C. We used PfRH1 (3D7ΔRH1), PfRH2a, and PfRH2b knockout mutants (3D7ΔRH2a and 3D7ΔRH2b) to compare the level of growth inhibition by selected human sera to the level of inhibition of 3D7 wild-type (3D7-WT) parasites, which express all PfRH1, PfRH2a, and PfRH2b antigens (1, 33).

### Growth inhibition assays

Growth inhibition assays of two cycles of replication were performed as described previously (51) using the 3D7 wild-type (3D7-WT) and PfRH knockout isolates. A greater inhibitory effect of antibodies on the wild-type lines would point towards the presence of inhibitory antibodies against PfRH antigens, providing insights about their role in antigenic variation and immune escape (7, 25, 26).

Briefly, parasites were synchronized using 5% D-sorbitol 30 hours before the assay. At mid-trophozoite stage, parasites were washed in RPMI HEPES and diluted to a final 1% hematocrit (using normal or neuraminidase treated RBCs) and a starting parasitemia of 0.1%–0.4%. Neuraminidase-treated erythrocytes were prepared the same day by incubating with 0.067 units/ml neuraminidase in RPMI-HEPES supplemented with 25mM NaHCO_3_ for 1 hour at 37˚C. Treated erythrocytes were washed three times and stored in RPMI-Hepes-NaHCO_3_. Parasites were aliquoted into sterile 96 well U-bottom plates and human sera were added in 1/10 dilution, avoiding the outer wells (filled with 200µl PBS) of each plate because of differential humidification. Plates were incubated in humidified and gassed boxes at 37˚C. After 40 hours, 5µl of culture media were added to each well. After 90 hours, plates were centrifuged (1200rpm, 1min at RT), washed twice with PBS, and finally resuspended in 100µl PBS containing 10µg/ml Ethidium Bromide stain (30–60 minutes in the dark). Pellets were resuspended in 200µl PBS and analyzed using flow cytometry. Parasitemia was counted as the percentage of trophozoite-infected red blood cells and expressed as the percentage of growth relative to a PBS control. All samples were tested in duplicate and expressed as the average and range of duplicates, unless otherwise stated.

To establish comparable invasion efficiency between the PfRH knockout mutants and the wild-type parasites, we tested the effect of known invasion inhibition reagents (unrelated to PfRH1 and PfRH2). Both heparin and the anti-merozoite antigen (AMA1)-binding peptide R1 had a similar inhibitory activity on 3D7 wild-type, 3D7ΔRH1, 3D7ΔRH2a, and 3D7ΔRH2b, confirming similar invasion efficiency of all lines (data not shown).

### Statistical analysis

Differential growth between the 3D7 wild-type (parental line) and knockout lines was defined as a >25% difference in relative growth (growth of knockout line divided by growth of parental wild-type line), on the basis that this difference was greater than the variance seen in the assays, and on the assumption that a difference of >25% was likely biologically significant. The cut-off for the absolute difference in growth was 10% (25, 26). Differences in median growth between knockout and wild type for inhibitor (defined as inhibition of wild-type more than 3D7ΔRH1 parasites by more than 25%) and non-inhibitor (with differential inhibition <=10%) samples were assessed using Mann–Whitney tests.

The correlation between IgG responses against PfRH antigen (OD) and invasion inhibition results was assessed by Spearman’s rank correlation. Differences in optical density (OD) between groups in the ELISAs were assessed using the Kruskal–Wallis test (for differences across age groups) and the Wilcoxon rank sum (Mann–Whitney) test for differences by parasitemic status.

## Results

### Antibodies to PfRH1 and PfRH2 by ELISA

**Figure 1:**
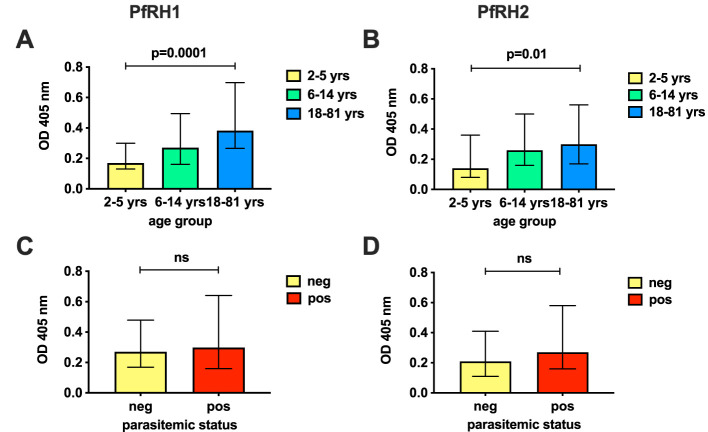
Antibodies to PfRh1 and PfRh2 in the study population. Prevalence of antibodies against PfRH1 **(A, C)** and PfRH2 **(B, D)** in Cohort 1 by ELISA (n=148). Responses have been classified by age **(A, B)** or parasitemic status **(C, D)** and show the median OD at 405nm and interquartile ranges of age groups 2–5, 6–14, and 18–81 years (yrs) **(A, B)**, or by parasitemic status by PCR (pos, positive; neg, negative; **C, D**). P-values indicate the significance of differences across the groups. Statistical significance was determined by the Kruskal–Wallis test (across age groups) and by the Wilcoxon rank sum (Mann–Whitney) test (by parasitemic status). The PfRH2 antigen used corresponded to a conserved region of PfRH2a and 2b. ns, non-significant.

**Figure 2:**
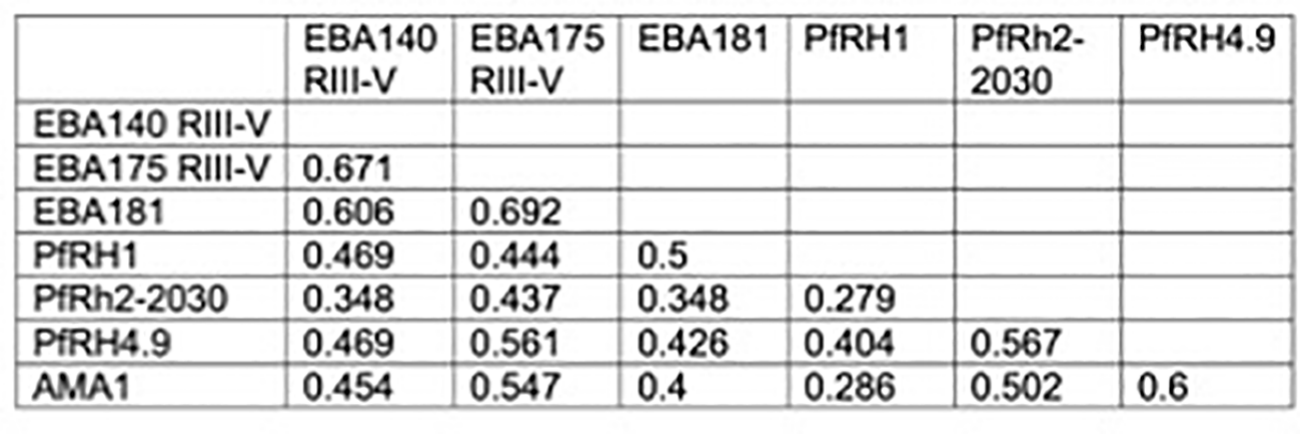
Relationship between PfRH1-specific antibodies and antibodies to other invasion ligands. Correlation between the presence of PfRH1-specific antibodies to different invasion ligands in Cohort 1. Values indicate Spearman’s r (rho) correlation (p<0.01 for all). RIII–V: regions III–V of EBA175 and EBA140, respectively. RH2-2030 covers a fragment in the common region of PfRH2a and PfRH2b. PfRH4.9: erythrocyte-binding region of PfRH4. PfRH1: erythrocyte-binding region.

First, we quantified antibodies to recombinant PfRH1 and PfRH2 proteins by ELISA in a cohort of children and adults in Cohort 1 (n=148, **Supplementary Table S1**). The results confirmed that both PfRH1 and PfRH2 were targets of acquired human antibodies in children and adults in our study populations (**Figure 1**). We observed an age-dependent acquisition of antibodies, reflected in higher median ODs in older children and adults (median OD of 0.17, 0.27, and 0.38 for age groups 2–5, 6–14, and 18–81 years, respectively, p=0.0001 for PfRH1; median OD 0.14, 0.26, and 0.3 for age groups 2–5, 6–14, and 18–81, respectively, p=0.01 for PfRH2, **Figures 1A, B**). Although the median ODs were higher in PCR-positive children, this did not reach statistical significance for either the PfRH1 or PfRH2 antigen (PfRH1: median OD of 0.27 versus 0.3; PfRH2: median OD of 0.21 versus 0.27 for parasite-negative and parasite-positive samples, respectively, **Figures 1C, D**). For comparison, we also measured antibodies to PfRH4 and members of the EBA invasion ligand family. Antibodies against PfRH1, PfRH2, and other merozoite invasion ligands such as PfRH4, EBA140, EBA175, EBA181, and AMA1 were moderately correlated, which suggested a co-acquisition of antibodies against multiple merozoite invasion ligands during exposure [**Figure 2** (26)].

### Differential inhibition of *P. falciparum* wild-type and PfRH1 knockout lines by naturally acquired antibodies.

**Figure 3:**
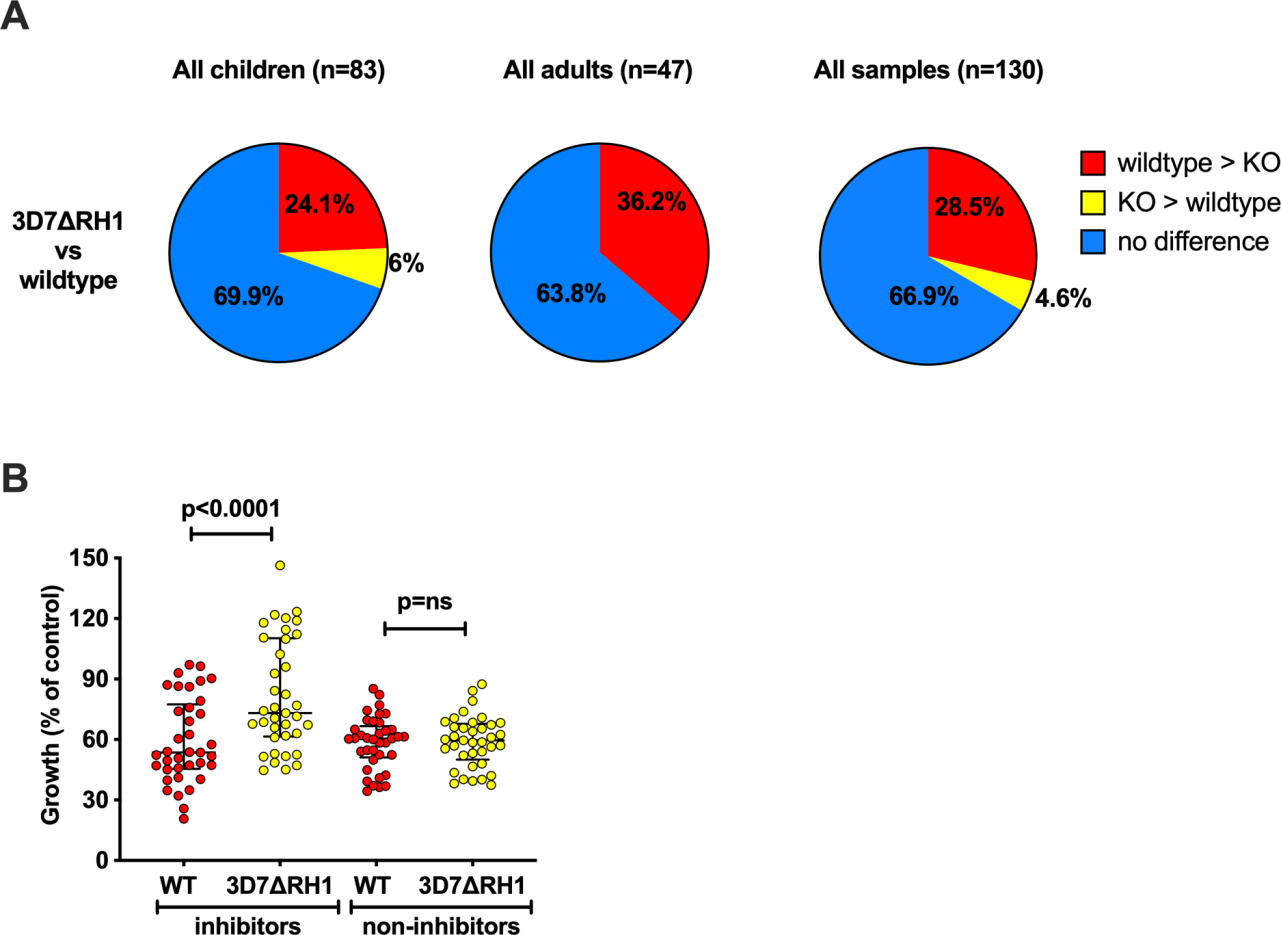
Differential inhibition of 3D7ΔRH1 vs wild-type (WT) parasites by naturally-acquired antibodies. **(A)** Proportion of samples that differentially inhibited the wild-type and knockout (KO) lines. Samples have been classified according to their inhibitory activity. Red, wild-type > KO: the wild-type more inhibited than knockout. Yellow, KO > wild-type: knockout more inhibited than wild-type. Blue, no difference: no differential inhibition between the two lines compared. **(B)** Inhibition of the wild-type and knockout *P. falciparum* lines by invasion profile. Samples were classified as inhibitors or non-inhibitors according to their inhibition of the wild-type, compared to 3D7ΔRH1 where the inhibitors inhibited the wild-type more than the knockouts with 25% difference or more in relative growth (indicating the presence of PfRH-specific antibodies), and the non-inhibitors showed 10% or less differential inhibition in either direction. Median growth of all categories was expressed as the percentage growth compared to a PBS control. Error bars show the interquartile range and p-values indicate levels of differences between the different lines tested (ns: not significant) as determined by paired t-tests.

To investigate the potential invasion-inhibitory function of PfRH1-specific antibodies and the role of PfRH1 in phenotypic variation and immune evasion, we compared the growth-inhibitory potential of serum antibodies in 71 randomly selected samples from malaria-exposed adults and children (Cohort 1) against 3D7-WT and 3D7ΔRH1 (**Figure 3A**). This knockout parasite line has been previously shown to not express PfRH1, and no upregulation in the transcription or expression of any other EBA or PfRH protein was observed (33). Hence, greater inhibition by antibodies of the 3D7-WT line compared to the 3D7ΔRH1 line may indicate the presence of PfRH1-specific inhibitory antibodies. In these assays, 13 samples (n=11 children; n=2 adults, **Supplementary Table S2**) out of 71 (18.3%) inhibited the wild-type parasites more than the knockout, suggesting the presence of PfRH1-specific inhibitory antibodies in these samples.

Since functional and quantitative studies suggested the presence of PfRH1-specific antibodies, we also examined the relationship between PfRH1 specific IgG in serum detected by ELISA using these 71 samples. There was no correlation between differential growth inhibitory activity and IgG reactivity (Spearman’s rho: r=0.06, p=0.69). The lack of correlation may be due to differences in the function of specific antibodies, i.e., not all PfRH-specific antibodies measured by ELISA contribute to growth inhibition as measured by GIA.

We further examined growth inhibition among children and adult residents from the same geographical region but at different times by also testing samples from Cohort 2 (children) and Cohort 3 (adults) (**Supplementary Table S1**). In total, 33.1% (n=43/130) of all samples tested (including n=71 from Cohort 1) showed differential growth inhibition of wild-type versus PfRH1 knockout parasites and 28.5% (37/130) of total samples inhibited 3D7-WT more than the PfRH1 knockout parasites; only 4.6% inhibited PfRH1 knockout more than 3D7-WT, (**Figure 3A**, third pie chart; **Supplementary Table S2**). These findings further suggest the presence of functional PfRH1-specific inhibitory antibodies and imply that upon disruption of *pfrh1*, the parasites relied on the use of other invasion ligands. PfRH1 is part of a repertoire of invasion ligands that can mediate invasion, and the loss of its expression may enable evasion of inhibitory antibodies, reflected in the lower inhibition of 3D7ΔRH1 compared to 3D7-WT. Only 4.6% (n=6/130) of the samples inhibited 3D7ΔRH1 more than wild-type parasites, possibly due to the presence of antibodies targeting ligands that were replacing the function of PfRH1 (**Supplementary Table S2**, **Figure 3A**, third pie chart).

To assess whether differentially inhibitory antibodies were acquired with age, we analyzed child and adult donor samples separately (**Figure 3A**, pie charts 1 and 2). The proportion of antibodies that inhibit the wild-type more than PfΔRH1 was higher in the adult samples compared to the children’s samples, consistent with the acquisition of antibodies with age and repeated exposure. Among the children’s samples (Cohort 1, n=52, and Cohort 2, n=31, total n=83), the overall proportion of differentially inhibitory samples was similar in the two different cohorts (14/52, 27% and 11/31, 35.5%, respectively, **Supplementary Table S2**). In both cohorts, a greater proportion of samples inhibited 3D7-WT more than 3D7ΔRH1 (21.2% (n=11/52) and 29% (n=9/31), respectively; **Supplementary Table S2**, rows 2 and 3). Among the adult samples (Cohort 1 n=19 and Cohort 3 n=28, total n=47), 36.2% (n=17/47) of samples inhibited the wild-type parasites more than 3D7ΔRH1 (**Supplementary Table S2**, rows 3 and 5; **Figure 3A**), suggesting the presence of inhibitory antibodies targeting PfRH1.

To further understand the magnitude of the difference in overall inhibition of 3D7-WT compared to 3D7ΔRH1 parasites by acquired antibodies, we compared the median growth of 3D7-WT and 3D7ΔRH1 when tested with samples that showed differential inhibitory activity (inhibitors) versus samples that did not show differential inhibition (non-inhibitors). This showed that the growth of 3D7ΔRH1 parasites was substantially higher (less inhibition) than the growth of wild-type parasites {median growth 73.1% [interquartile ranges (IQR) 61.5%–110.2%] compared to 53.6% [IQR 45.5%–77.5%], respectively, p<0.0001; **Figure 3B**}. The difference between wild-type and 3D7ΔRH1 was not significant when comparing the median growth effect of samples that were not differentially inhibitory (**Figure 3B**). These data further support the conclusion that PfRH1 is a significant target of growth inhibitory antibodies among some individuals.

### Acquired antibodies differentially inhibit wild-type and PfRH2 knockout lines

**Figure 4:**
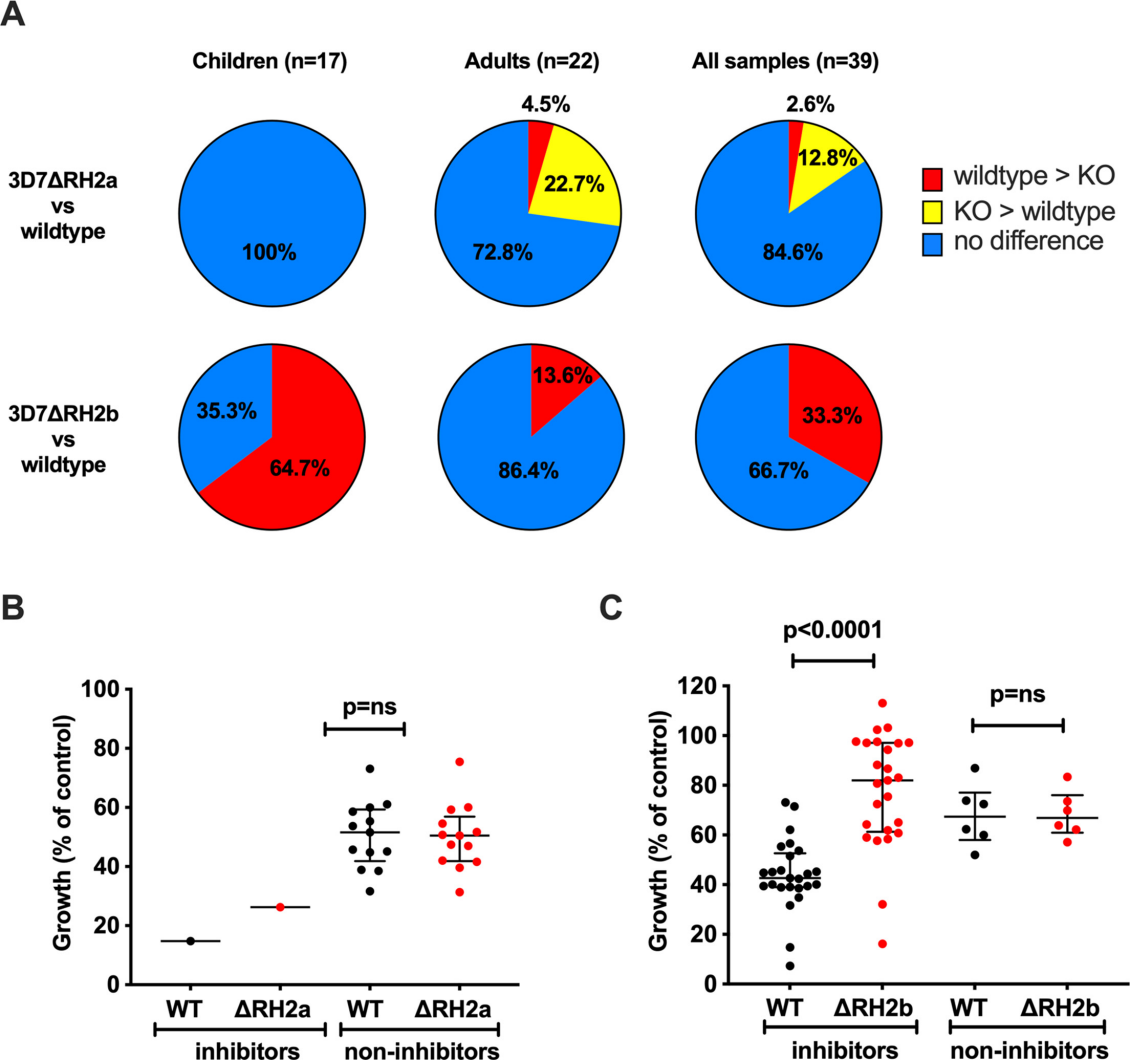
Differential inhibition of 3D7ΔRH2a and 3D7ΔRH2b vs wild-type (WT) parasites by naturally-acquired antibodies. **(A)** Proportion of samples that differentially inhibited the wild-type and knockout (KO) lines. Samples have been classified according to their inhibitory activity. Red, wild-type > KO: wild-type more inhibited than knockout. Yellow, KO > wild-type: knockout more inhibited than the wild-type. Blue, no difference: no differential inhibition between the two lines compared. **(B, C)** Inhibition of the wild-type and knockout *P. falciparum* lines by invasion profile. Samples were classified according to their inhibition of the wild-type compared to 3D7ΔRH2a **(B)** and 3D7ΔRH2b **(C)**. Samples were classified as inhibitors and non-inhibitors, where the inhibitors inhibited the wild-type more than the knockouts with 25% difference or more in relative growth (indicating the presence of PfRH-specific antibodies), and the non-inhibitors showed 10% or less differential inhibition in either direction. Median growth of all categories was expressed as the percentage growth compared to a PBS control. Error bars show the interquartile range and p-values indicate levels of differences between the different lines tested (ns: not significant) as determined by paired t-tests.

To investigate the potential functional effect of PfRH2-specific antibodies, or whether loss of PfRH2a or 2b alters susceptibility to inhibitory antibodies, we also compared the inhibition of 3D7-WT with 3D7ΔRH2a and 3D7ΔRH2b knockout lines using cohort sera samples. A subset of 39 sera from children and adults (17 children’s samples from Cohort 2, 22 adult samples from Cohort 3, **Supplementary Table S1**) were assessed. Among all the samples tested (n=39), we observed differential inhibitory effects on 3D7 wild-type compared to 3D7ΔRH2a and on 3D7 wild-type compared to 3D7ΔRH2b (**Figure 4A**, **Supplementary Table S3**). Greater inhibition by antibodies to the 3D7-WT line compared to the 3D7ΔRH2 line may indicate the presence of PfRH2-specific inhibitory antibodies.

Comparing the inhibitory effects between wild-type parasites and 3D7ΔRH2a, 15.4% (n=6/39) of all samples showed a differential inhibitory effect (**Figure 4A**, row 1, last pie chart). Only one sample (1/39, 2.6%) inhibited wild-type parasites (which express both PfRH2a and PfRH2b) more than 3D7ΔRH2a, suggesting a limited presence of PfRH2a-specific inhibitory antibodies. Interestingly, 12.8% (n=5/39) inhibited 3D7ΔRH2a more than the wild-type parasites (**Figure 4A**, row 1, last pie chart, **Supplementary Table S3**), suggesting that the loss of PfRH2a rendered the parasites more susceptible to the inhibitory effect of antibodies targeting other invasion ligands.

In contrast to results shown using 3D7ΔRH2a, 33.3% (n=13/39) of samples inhibited wild-type parasites more than 3D7ΔRH2b, suggesting the presence of PfRH2b-specific inhibitory antibodies in these sera. We did not observe greater inhibition of 3D7ΔRH2b compared to wild-type parasites by any samples (**Figure 4A**, row 2, last pie chart, **Supplementary Table S3**). These results suggest that variation in the expression or utilization of PfRH2b may facilitate evasion of inhibitory antibodies, with loss of PfRH2b leading to reduced antibody inhibition. These findings demonstrate the different phenotypes between PfRH2a- and PfRH2-knockout lines and suggest that the two PfRH2 ligands may have different functions and are targeted differently by acquired antibodies.

When we compared the inhibitory effect between the two knockout lines, 48.8% (n=19/39) of the samples inhibited the 3D7ΔRH2a (RH2b-positive) more than the 3D7ΔRH2b (RH2a-positive) parasites, further suggesting the presence of PfRH2b-specific inhibitory antibodies, and a limited presence of PfRH2a-specific inhibitory antibodies (**Supplementary Table S3**). This supports the hypothesis that the PfRH2a- and PfRH2b-knockout lines are antigenically different, with PfRH2a playing a minor role as a target of inhibitory antibodies.

To understand the magnitude of the difference in overall inhibition, we compared the median growth of 3D7-WT and 3D7ΔRH2a/b when tested with samples that showed differential inhibitory activity (inhibitors) versus samples that did not show differential inhibition (non-inhibitors). This showed that the growth of 3D7ΔRH2b parasites was substantially higher (less inhibition) than the growth of wild-type parasites, but this difference was not seen for 3D7ΔRH2a (**Figure 4B, C**); wild-type parasites were more inhibited than 3D7ΔRH2b [median growth was 82% (IQR 61.3%–97.1%) for PfRH2b-knockout parasites versus 42.7% (39%–52.6%) for 3D7-WT parasites (p=0.0001)]. These findings provide further evidence that PfRH2b is a substantial target of inhibitory antibodies among some individuals, whereas PfRH2a is not a major target.

We investigated the relationship between the PfRH2-specific antibodies by ELISA and differential inhibition of PfRH2a- and PfRH2b-knockout parasites using the subset of samples (n=39) from children and adults (Cohorts 1 and 3). We did not observe a significant correlation between the presence of antibodies by ELISA and inhibitory activity (Spearman’s r= -0.01; p=NS).

### Relatedness of inhibitory responses to PfRH and EBA ligands

**Figure 5:**
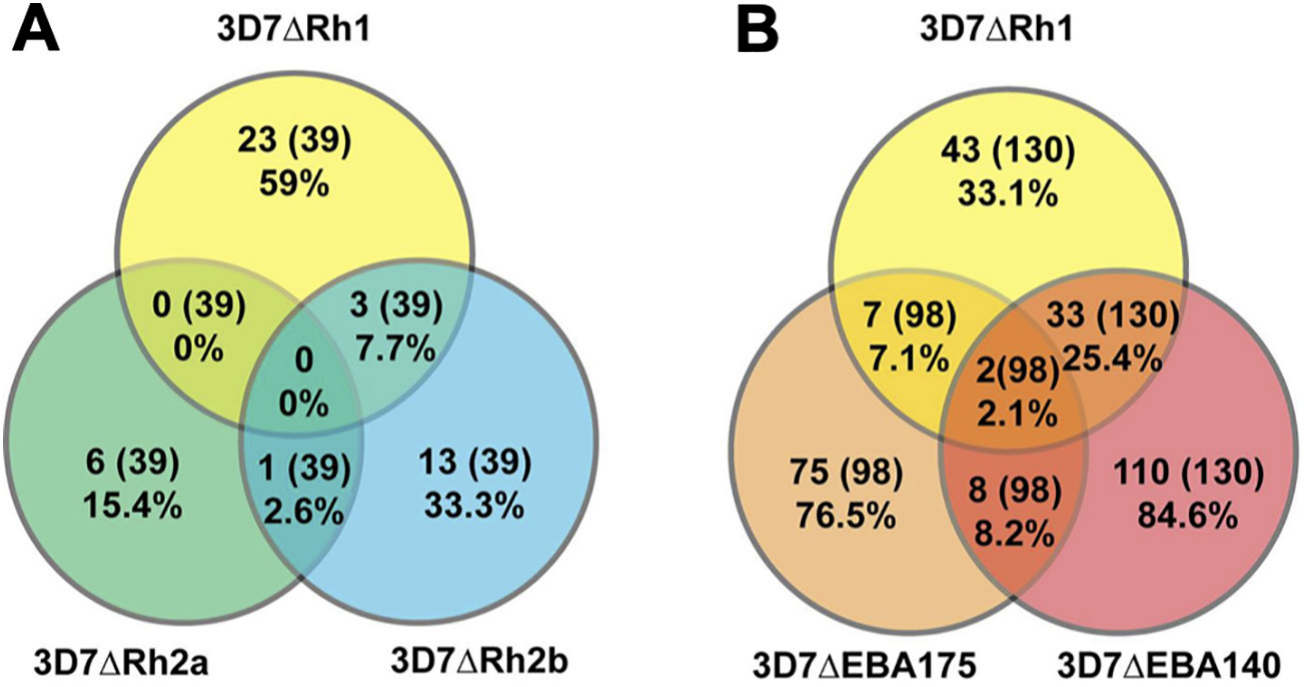
Relatedness in the inhibitory activity against different *P. falciparum* lines. Proportion of differentially inhibitory samples that inhibited PfRH1 and PfRH2a- or PfRH2b-knockout lines **(A)**, or PfRH1 and EBA140- or EBA175-knockout lines **(B)** compared to the parental line (both directions of differential inhibition have been included: the wild-type more inhibited than knockout, and knockout more inhibited than the wild-type). The proportion is expressed as the percentage of the total number of samples that were tested against the two lines being compared as well as the wild-type parasites. The values in the overlaps show the proportion of all samples tested that differentially inhibited the compared lines in the same direction.

**Figure 6:**
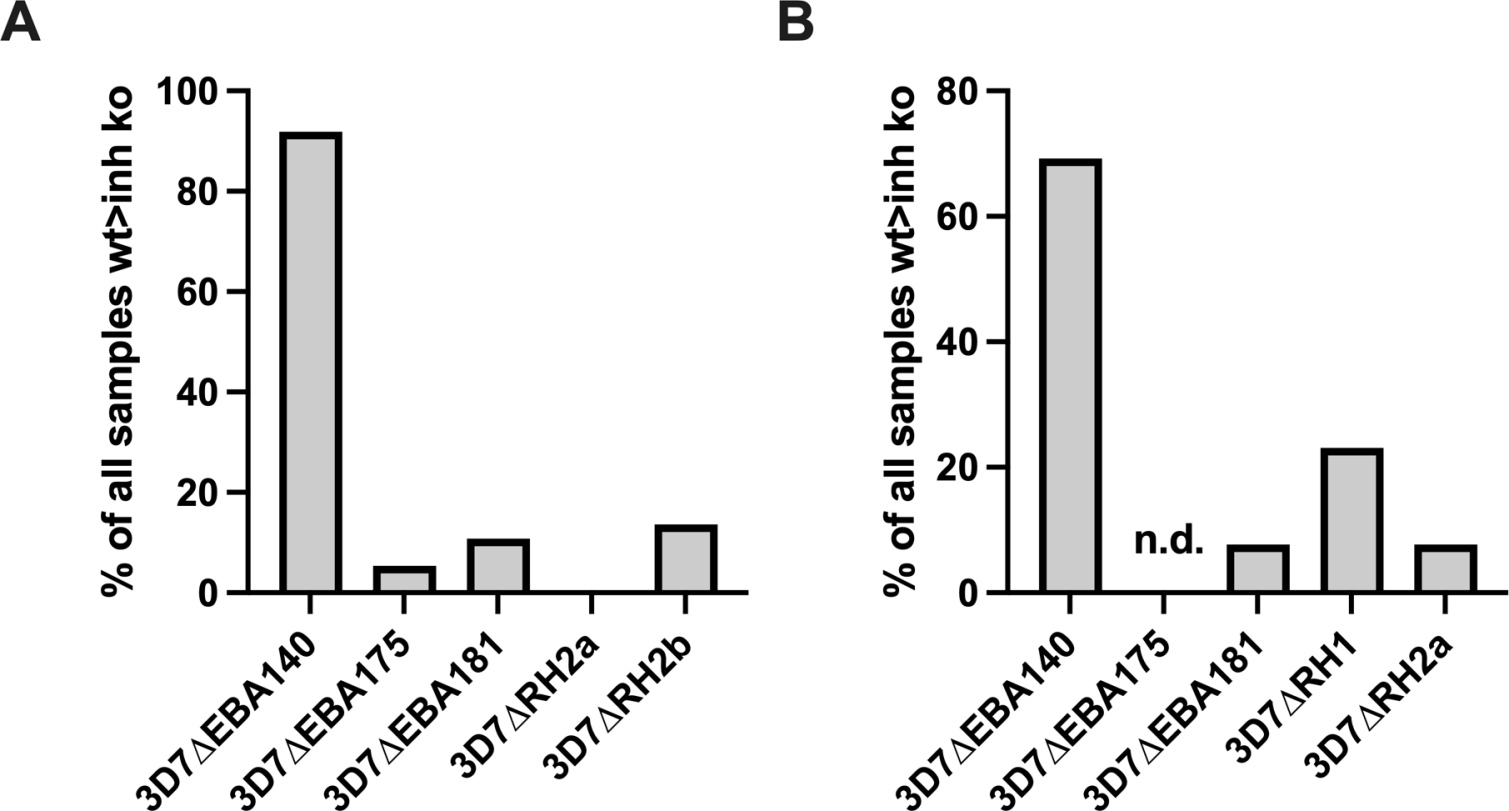
Proportion of samples with broader inhibitory activity. Samples that inhibited the wild-type more than 3D7ΔRH1 **(A)**, or more than 3D7ΔRH2b **(B)**, were assessed for whether they also inhibited the wild-type more than other knockout lines, including 3D7ΔEBA140, 3D7ΔEBA175, 3D7ΔEBA181, or 3D7ΔRh2a. The number of samples that inhibited the wild-type more than 3D7ΔRH1 **(A)** or 3D7ΔRH2b **(B)** and an additional knockout line (as shown on x-axis) is expressed as the percentage of all samples that inhibited the wild-type more than the 3D7ΔRh1 **(A)** or 3D7RH2b knockouts **(B)**. The proportion of samples that inhibited the wild-type more than EBA140-KO parasites was significantly higher (p<0.001) than observed with other KO parasite lines in **(A, B)**.

To better understand the antigenic differences between the different PfRH-knockout lines, we examined the degree of overlap between samples that differentially inhibited the wild-type and the different knockout lines (**Figures 5**, **6**, **Supplementary Table S4**).

#### PfRH1 vs PfRH2 inhibitory responses

We compared the differential invasion profiles of samples against 3D7ΔRH1, 3D7ΔRH2a, or 3D7ΔRH2b parasites versus wild-type (**Figure 5A**, **6A**). Only 7.7% of all samples tested showed the same inhibition profile against both 3D7ΔRH1 and 3D7ΔRH2b (**Figure 5A**) (3/39; where 39 is the number of all samples that were tested against 3D7ΔPfRH1 and 3D7ΔPfRH2b parasites versus wild-type). None of these samples showed the same inhibition profile for 3D7ΔRH1 and 3D7ΔRH2a. Among the samples that inhibited the wild-type more than the 3D7ΔRH1 line, only 13.6% of samples also inhibited the wild-type more than the 3D7ΔRH2b line (3/22 samples; where 22 is the number of samples that inhibited the wild-type more than 3D7ΔPfRH1) (**Figure 6A**). These differences in susceptibility to inhibitory antibodies indicate that there are distinct antigenic differences between 3D7ΔRH1 and 3D7ΔRH2b parasites, and that inhibitory antibodies target different antigens in 3D7ΔRH1 and 3D7ΔRH2b parasites.

#### PfRH1 vs. EBA inhibitory responses

We also compared inhibitory profiles between the PfRH1 knockout line with EBA knockout lines (**Figures 5**, **6**). Remarkably, of the 37 samples that inhibited the wild-type more than 3D7ΔRH1, 34 (91.9%) samples also inhibited the wild-type more than the 3D7ΔEBA140 line (**Figure 6A**). This concordance of results suggests either similar antigenicity or, alternatively, that antibodies target similar invasion ligands in both the 3D7ΔRH1 and 3D7ΔEBA140 parasite lines. In contrast, only two (5.4%) and four (10.8%) samples also inhibited the wild-type more than 3D7ΔEBA175 and 3D7ΔEBA181, respectively [refer to (26) for 3D7ΔEBA results], indicating that the 3D7ΔRH1 phenotype differed from the 3D7ΔEBA175 and 3D7ΔEBA181 phenotype. Of all samples tested, 34 out of 130 (26.2%) inhibited the wild-type more than both 3D7ΔRH1 and ΔEBA140. In contrast, only 2 out of 98 (2.0%) and 4 out of 130 (3.1%) had the same differential inhibitory effect on 3D7ΔRH1 versus the wild-type and ΔEBA175 versus the wild-type or 3D7ΔEBA181 versus the wild-type, respectively, suggesting that these knockout lines were antigenically different.

#### PfRH2 vs. EBA inhibitory responses

When we compared the relatedness of responses between 3D7ΔRH2a/2b and EBA knockouts versus the wild-type (**Figure 6B**), we observed a concordance between 9 out of 13 samples (69.2%) that inhibited the wild-type more than 3D7ΔRH2b and also inhibited the wild-type more than 3D7ΔEBA140 (**Figure 6B**), pointing towards some antigenic relatedness or overlap in antibody activities between 3D7ΔRH2b and 3D7ΔEBA140. Of the total samples tested against both lines, 9 out of 39 samples (23.1%) had the same inhibitory profile against both knockouts versus wild-type combinations. In contrast, there was very limited relatedness between 3D7ΔRH2b and 3D7ΔEBA181. There was also no strong relationship between the differential inhibition of 3D7ΔRH2a and any of the 3D7ΔEBA- or 3D7ΔRH-knockout lines (**Figures 5**, **6**; **Supplementary Table S4**).

Overall, few samples inhibited the KO parasites more than the wild-type parasites (5/98 samples, 5.1%; 1/130 samples, 0.8%; and 4/130, 3.1% for comparisons between 3D7ΔEBA175 vs WT, 3D7ΔEBA140 vs WT, 3D7ΔEBA181 vs WT and 3D7ΔRH1 vs WT, respectively, **Supplementary Table S4**). Comparing the relatedness of responses in these cases, there were five out of six (83.3%), one out of six (16.7%), and four out of six (66.7%) samples, respectively, that inhibited 3D7ΔRH1 more than the wild-type and inhibited 3D7ΔEBA175, 3D7ΔEBA140, and 3D7ΔEBA181 more than the wild-type. Taken together, these results suggested that although there was some overlap in responses against certain knockout lines (i.e., 3D7ΔRH1 and 3D7ΔEBA140 as well as 3D7ΔRH2b and 3D7ΔEBA140), most invasion phenotypes were antigenically distinct and contributed to the antigenic diversity of the parasites.

## Discussion

This study aimed to determine the role of PfRH1, PfRH2a, and PfRH2b as targets of invasion inhibitory antibodies and their potential role in immune evasion, mediated by the variation in their expression, which is known to occur with clinical and laboratory isolates (7, 8). We used parasite lines with disrupted expression of PfRH1, PfRH2a, or PfRH2b to measure the inhibitory effect of antibodies on these lines in comparison to the wild-type parental line. We found differential inhibition of the wild-type parasites compared to the knockout lines, supporting the presence of PfRH-specific inhibitory antibodies. Strikingly, we found that over a third of samples from *P. falciparum*-exposed individuals differentially inhibited the 3D7ΔRH1, 3D7ΔRH2b, and wild-type parasites, indicating that the differential expression or use of PfRH invasion ligands may facilitate evasion of inhibitory antibodies. To date, the role of PfRH2a in invasion has been unclear. Our study showed limited evidence of the presence of inhibitory antibodies against PfRH2a, pointing towards this invasion ligand as playing only a minor role (1). Furthermore, when comparing the inhibitory potential of samples against 3D7ΔRH2a and 3D7ΔRH2b, 41% of samples inhibited the 3D7ΔRH2a parasites (still expressing 3D7RH2b) more than the 3D7ΔRH2b parasites, reinforcing that PfRH2b and PfRH2a may be major and minor targets of inhibitory antibodies, respectively.

Members of the EBA family of invasion ligands have previously been identified as targets of invasion inhibitory antibodies and mediators of immune evasion (25, 26). When examining the relatedness of the inhibitory responses, we only found limited overlap, suggesting that each invasion ligand is associated with a specific antigenic profile. Using knockout lines as a means of disrupting the expression of an invasion ligand may mimic switches that occur to different invasion ligands and pathways among clinical isolates, thus changing the antigenic profile. Using this approach, and together with data from previous EBA knockout studies, we conclude that both the EBA and PfRH families of invasion ligands are targets of invasion inhibitory antibodies and that switching between invasion pathways and variation in the expression and use of those ligands facilitates immune evasion.

The ELISA data showed that, in our study population, antibodies against PfRH1 and PfRH2 were readily acquired in an age-dependent pattern, suggesting a potential role of acquired immunity. The lack of clear correlations between the presence of antibodies by ELISA and ligand-specific invasion inhibition in our study has been previously observed for other invasion ligands (26, 52). This may in part be because ELISAs use truncated proteins to assess antibody presence, and therefore, antibodies against epitopes present in other parts or domains of the protein are not measured. In parasite inhibition assays, merozoites present full-length antigens in their natural conformation and molecular environment, which may lead to different accessibility by antibodies compared to antibodies quantified by ELISA using recombinant proteins. Furthermore, antibody subclasses, affinity and avidity, and fine specificity are not measured in standard ELISAs but might play an important role in the mode of functional activity and/or protection. Additionally, as antibodies to multiple ligands are co-acquired with malaria exposure and antibodies with multiple specificities are present in serum samples, it is difficult to dissect the relationship between a specific response measured by an ELISA and invasion inhibitory antibodies. The advantage of our *in vitro* approach using live parasites is the presentation of the whole antigen in its correctly folded state as it occurs in live parasites. Since acquired immunity appears to be a complex interplay of various antibody effector mechanisms, more functional assays need to be conducted to fully understand the role of invasion inhibitory antibodies in acquired immunity. Furthermore, studies of B cell memory for PfRh proteins could be valuable for understanding long-term immunity.

Our results provide evidence that PfRH1 and PfRH2 are targeted by invasion inhibitory antibodies and further support that *P. falciparum* evades the host’s immune response by switching between invasion pathways and ligand use. The use of different ligand-receptor interactions is ultimately defined by a range of factors, such as strain-specific preferences; the genetic make-up of a given parasite strain, ligand, and receptor sequence polymorphism; the degree of host immunity; and possibly others (3). Current knowledge does not suggest there is complex formation between different PfRH proteins. Therefore, differential inhibition of wild-type versus PfRH KO parasites likely reflects the presence of antibodies to specific PfRH proteins rather than antibodies to epitopes formed by PfRH complexes. It has also been suggested that not all EBA and PfRH invasion pathways are interchangeable and may not be strictly redundant (3); therefore, more insight is needed into the complexity and individual relevance of the various invasion pathways to understand how invasion ligands could be targeted in vaccine development. Optimal naturally acquired immunity to malaria is defined by antibodies to multiple antigens (14). To achieve high-level efficacy, it is likely that a blood-stage vaccine must target several ligands of the invasion machinery, or alternatively, invasion ligands of the EBA and/or PfRH family in combination, together with critical bottleneck ligands such as PfRH5 or AMA1.

In summary, we provide evidence that invasion ligands PfRH1 and PfRH2 are part of the repertoire of invasion ligands that is targeted by acquired human antibodies and their variation in expression or use may contribute to immune evasion. While antibodies to PfRH1 and PfRH2 inhibit the invasion of erythrocytes by merozoites directly, there may also be other effector mechanisms that have not yet been fully characterized. Further research is needed to identify the most promising combination of merozoite antibodies to inform blood-stage vaccine development.

## Data Availability

Data that were analysed in the preparation of this paper and presented in figures and tables are available from the corresponding author for non-commercial purposes, pending agreement from relevant ethics committees. *P. falciparum* sequences were obtained from the public database at PlasmoDB.org.
