# Supplementary material for: Plasmodium falciparum reticulocyte-binding homologues are targets of human inhibitory antibodies and play a role in immune evasion
Source: Front Immunol. 2025 Mar 25;16:1532451. doi: 10.3389/fimmu.2025.1532451 (PMC11975925; doi:10.3389/fimmu.2025.1532451)
Supplement: Supplementary file 1 [file Table1.docx]

**Supplementary table S1**

**Samples tested on respective parasite lines**

|  | Cohort 1 (total, n=71): Children (n=52),  Adults (n=19) | Cohort 2  Children  (n=31) | Cohort 3  Adults  (n=28) |
| --- | --- | --- | --- |
| 3D7∆RH1 vs wt^1^ | 71 | 31 | 28 |
| 3D7∆RH2a vs wt^1^ | n.d.^2^ | 17 | 22 |
| 3D7∆RH2b vs wt^1^ | n.d.^2^ | 17 | 22 |
| ELISA PfRH1 | All 148 | n.d.^2^ | n.d.^2^ |
| ELISA PfRH2 | All 148 | n.d.^2^ | n.d.^2^ |

Notes:

^1^ vs wt: versus wildtype

^2^ n.d.: not done

**Supplementary Table S2**

**Proportion of samples with differential inhibitory properties: PfRH1**

| Sample set | Differential  inhibition | n (%) |
| --- | --- | --- |
| 1. **All samples (n=130)** | **wildtype > KO**  **KO > wildtype** | **37 (28.5%)**  **6 (4.6%)**  **43 (33.1%)** |
| 1. Cohort 1:   Ng children 1998 (n=52) | wildtype > KO  KO > wildtype | 11 (21.2%)  3 (5.8%)  14 (27%) |
| 1. Cohort 1:   Ng adults 1998 (n=19) | wildtype > KO  KO > wildtype | 2 (10.5%)  0 (0%)  2 (10.5%) |
| 1. Cohort 2:   Ng children 2003 (n=31) | wildtype > KO  KO > wildtype | 9 (29.0%)  2 (6.5%)  11 (35.5%) |
| 1. Cohort 3:   Kilifi adults 2004 (n=28) | wildtype > KO  KO > wildtype | 15 (53.6%)  0 (0%)  15 (53.6%) |

Notes:

Number (n) and percentage (%) of samples with differential inhibitory activity on 3D7∆RH1 and 3D7 wildtype are shown by cohort. Samples were classified according to inhibitory activity. Wildtype > KO: wildtype was more inhibited than 3D7∆RH knockout1; KO > wildtype: 3D7∆RH1 knockout was more inhibited than wildtype. Definition of differential inhibition was >25% difference in relative growth.

**Supplementary Table S3**

**Proportion of samples with differential inhibitory properties: PfRH2**

| Sample set | Differential  inhibition | 3D7∆RH2a  vs  wildtype | | 3D7∆RH2b  vs  wildtype | | 3D7∆RH2a  vs  3D7∆RH2b | |
| --- | --- | --- | --- | --- | --- | --- | --- |
| **Cohort 2:**  **All samples**  **Ng 2003 (n=39)** | wildtype > KO  KO > wildtype | | **1 (2.6%)**  **5 (12.8%)** | | **13 (33.3%)**  **0 (0%)** | | n.a.  n.a. |
|  | ∆a > ∆b  ∆b > ∆a | | n.a.  n.a. | | n.a.  n.a. | | **19 (48.8%)**  **0 (0%)** |
| **Cohort 2:**  **Ng children 2003 (n=17)** | wildtype > KO  KO > wildtype | | **0 (0%)**  **0 (0%)** | | **11 (64.7%)**  **0 (0%)** | | n.a.  n.a. |
|  | ∆a > ∆b  ∆b > ∆a | | n.a.  n.a. | | n.a.  n.a. | | **10 (58.8%)**  **0 (0%)** |
| **Cohort 3:**  **Kilifi adults (n=22)** | wildtype > KO  KO > wildtype | | **1 (4.5%)**  **5 (22.7%)** | | **3 (13.6%)**  **0 (0%)** | | n.a.  n.a. |
|  | ∆a > ∆b  ∆b > ∆a | | n.a.  n.a. | | n.a.  n.a. | | **9 (40.9%)**  **0 (0%)** |

Notes:

Number (n) and percentage (%) of samples with differential inhibitory activity on 3D7 wildtype, 3D7∆RH2a and 3D7∆RH2b. Samples were classified according to inhibitory activity. Comparisons between 3D7∆RH2a and wildtype, 3D7∆RH2b and wildtype, and 3D7∆RH2a and 3D7∆RH2b are shown. Wildtype > KO: wildtype was more inhibited than knockout; KO > wildtype: knockout was more inhibited than wildtype. ∆a>∆b: 3D7∆RH2a was more inhibited than 3D7∆RH2b. ∆b>∆a: 3D7∆RH2b was more inhibited than 3D7∆RH2a. Definition of differential inhibition was >25% difference in relative growth.

**Supplementary table S4**

**Relatedness of responses**

| Reference line | compared to | KO > wildtype* | | wildtype > KO* | | KO > wildtype ^ | | wildtype > KO ^ | |
| --- | --- | --- | --- | --- | --- | --- | --- | --- | --- |
|  |  | n | % | n | % | n | % | n | % |
| 3D7∆RH1 | 3D7∆EBA175 | 5/6 | 83.3% | 2/37 | 5.4% | 5/98 | 5.1% | 2/98 | 2.0% |
|  | 3D7∆EBA140 | 1/6 | 16.7% | **34/37** | **91.9%** | 1/130 | 0.8% | 34/130 | 26.2% |
|  | 3D7∆EBA181 | 4/6 | 66.7% | 4/37 | 10.8% | 4/130 | 3.1% | 4/130 | 3.1% |
|  | 3D7∆RH2a | 0/2 | 0% | 0/22 | 0% | 0/39 | 0% | 0/39 | 0% |
|  | 3D7∆RH2b | 0/2 | 0% | 3/22 | 13.6% | 0/39 | 0% | 3/39 | 7.7% |
|  |  |  |  |  |  |  |  |  |  |
| 3D7∆RH2a | 3D7∆Eba175 | n.a.^1^ | n.a.^1^ | n.a.^1^ | n.a.^1^ | 0/19 | 0% | 0/19 | 0% |
|  | 3D7∆EBA140 | 0/5 | 0% | 0/1 | 0% | 0/39 | 0% | 0/39 | 0% |
|  | 3D7∆EBA181 | 0/5 | 0% | 1/1 | 100% | 0/39 | 0% | 1/39 | 2.6% |
|  | 3D7∆RH1 | 0/5 | 0% | 0/1 | 0% | 0/39 | 0% | 0/39 | 0% |
|  | 3D7∆RH2b | 0/5 | 0% | 1/1 | 100% | 0/39 | 0% | 1/39 | 2.6% |
|  |  |  |  |  |  |  |  |  |  |
| 3D7∆RH2b | 3D7∆Eba175 | n.a.^1^ | n.a.^1^ | n.a.^1^ | n.a.^1^ | 0/19 | 0% | 0/19 | 0% |
|  | 3D7∆EBA140 | 0/0 | 0% | 9/13 | 69.2% | 0/39 | 0% | 9/39 | 23.1% |
|  | 3D7∆EBA181 | 0/0 | 0% | 1/13 | 7.7% | 0/39 | 0% | 1/39 | 2.6% |
|  | 3D7∆RH1 | 0/0 | 0% | 3/13 | 23.1% | 0/39 | 0% | 3/39 | 7.7% |
|  | 3D7∆RH2a | 0/0 | 0% | 1/13 | 7.7% | 0/39 | 0% | 1/39 | 2.6% |

Notes:

Comparison of inhibitory effect of samples on different knock-out lines to assess relatedness of responses.

**Grey column 1**: Knockout strains 3D7∆RH1, 3D7∆RH2a or 3D7∆RH2b compared to other knockout lines (**grey column 2)**.

**Blue columns**: * compares only the samples that showed differential inhibition on 3D7∆RH1, 3D7∆RH2a or 3D7∆RH2b compared to wildtype, respectively (grey column 1), with inhibition profile of samples on other knockout versus wildtype comparisons (grey column 2). *For example (row 1): n=37 samples inhibited the wildtype more than the 3D7∆Rh1 knockout; out of these 37 samples, only 2 samples (5.4%) also inhibited the wildtype more than the 3D7∆EBA175 knockout.*

**Yellow columns**: ^ compares all samples tested. *For example (row 1): n=98 samples were tested on the wildtype vs knockouts, and 2 samples had an inhibitory effect on both wildtype vs 3D7∆RH1 and also on wildtype vs 3D7∆EBA175.*

KO > wildtype: knockout line was more inhibited than wildtype line

wildtype > KO: wildtype more inhibited than knockout line

^1^ Samples inhibitory on 3D7∆RH2a and 3D7∆RH2b haven’t been tested on 3D7∆EBA175
